# Ultrapotent human antibodies protect against SARS-CoV-2 challenge via multiple mechanisms

**DOI:** 10.1126/science.abe3354

**Published:** 2020-09-24

**Authors:** M. Alejandra Tortorici, Martina Beltramello, Florian A. Lempp, Dora Pinto, Ha V. Dang, Laura E. Rosen, Matthew McCallum, John Bowen, Andrea Minola, Stefano Jaconi, Fabrizia Zatta, Anna De Marco, Barbara Guarino, Siro Bianchi, Elvin J. Lauron, Heather Tucker, Jiayi Zhou, Alessia Peter, Colin Havenar-Daughton, Jason A. Wojcechowskyj, James Brett Case, Rita E. Chen, Hannah Kaiser, Martin Montiel-Ruiz, Marcel Meury, Nadine Czudnochowski, Roberto Spreafico, Josh Dillen, Cindy Ng, Nicole Sprugasci, Katja Culap, Fabio Benigni, Rana Abdelnabi, Shi-Yan Caroline Foo, Michael A. Schmid, Elisabetta Cameroni, Agostino Riva, Arianna Gabrieli, Massimo Galli, Matteo S. Pizzuto, Johan Neyts, Michael S. Diamond, Herbert W. Virgin, Gyorgy Snell, Davide Corti, Katja Fink, David Veesler

**Affiliations:** 1 Department of Biochemistry, University of Washington, Seattle, WA 98195, USA.; 2 Institut Pasteur and CNRS UMR 3569, Unité de Virologie Structurale, Paris, France.; 3 Humabs BioMed SA, a subsidiary of Vir Biotechnology, Bellinzona, Switzerland.; 4 Vir Biotechnology, San Francisco, CA 94158, USA.; 5 Departments of Medicine, Molecular Microbiology, Pathology and Immunology, Washington University School of Medicine, St. Louis, MO, USA.; 6 Rega Institute for Medical Research, Laboratory of Virology and Chemotherapy, KU Leuven, Belgium.; 7 III Division of Infectious Diseases, Luigi Sacco University Hospital, University of Milan, Italy.; 8 Washington University School of Medicine, St. Louis, MO, USA.; 9 UTSouthwestern Medical Center, Dallas, TX, USA.

## Abstract

Efficient therapeutic options are needed to control the spread of severe acute respiratory syndrome coronavirus 2 (SARS-CoV-2) that has caused more than 922,000 fatalities as of 13 September 2020. We report the isolation and characterization of two ultrapotent SARS-CoV-2 human neutralizing antibodies (S2E12 and S2M11) that protect hamsters against SARS-CoV-2 challenge. Cryo–electron microscopy structures show that S2E12 and S2M11 competitively block angiotensin-converting enzyme 2 (ACE2) attachment and that S2M11 also locks the spike in a closed conformation by recognition of a quaternary epitope spanning two adjacent receptor-binding domains. Antibody cocktails that include S2M11, S2E12, or the previously identified S309 antibody broadly neutralize a panel of circulating SARS-CoV-2 isolates and activate effector functions. Our results pave the way to implement antibody cocktails for prophylaxis or therapy, circumventing or limiting the emergence of viral escape mutants.

## A strong cocktail against SARS-CoV-2

Severe acute respiratory syndrome coronavirus 2 (SARS-CoV-2) infection is initiated by the trimeric spike protein that decorates the virus and binds the ACE2 receptor. Antibodies against the spike that neutralize viral infection have potential as therapeutics. Tortorici *et al.* describe two very potent antibodies, S2E12 and S2M11. Electron microscopy structures characterized the binding and showed that S2E12 traps the spike in a conformation that cannot bind ACE2. Both antibodies protected hamsters against SARS-CoV-2 challenge and may be useful in antibody cocktails to combat the virus and prevent the development of resistance.

*Science*, this issue p. 950

## Teaser

A potent antibody cocktail blocks attachment of SARS-CoV-2 to the host receptor and activates a protective immune response.

Severe acute respiratory syndrome coronavirus 2 (SARS-CoV-2) emerged at the end of 2019 and was sequenced by January 2020 (*1*, *2*). Although the reservoir host responsible for spillover into the human population remains uncertain, SARS-CoV-2 appears to have originated in bats from which closely related viruses and viral sequences have been identified (*1*, *3*). SARS-CoV-2 belongs to the sarbecovirus subgenus and is closely related to SARS-CoV, which was responsible for an epidemic in 2002–2003 that resulted in 8098 cases and 774 fatalities worldwide (*4*, *5*). The lack of preexisting immunity to SARS-CoV-2 due to its divergence from the four circulating endemic coronaviruses, and its high human-to-human transmissibility, have resulted in the ongoing coronavirus disease 2019 (COVID-19) pandemic, which has already caused more than 29 million infections and more than 922,000 fatalities as of mid-September 2020.

SARS-CoV-2 infection is initiated upon attachment of the viral transmembrane spike (S) glycoprotein via a receptor-binding motif (RBM) to angiotensin-converting enzyme 2 (ACE2), leading to membrane fusion and entry into host cells (*6*–*13*). As for all coronaviruses, SARS-CoV-2 S is the main target of neutralizing antibodies (Abs) and a focus of vaccine design and therapeutic targeting efforts (*14*). Although vaccine development programs are fast-tracked (*15*–*20*), large-scale manufacturing and administration to a large enough population for achieving community protection will likely take many months. Prophylactic and/or therapeutic antiviral drugs could address the gap before safe and efficient vaccines become widely available and will continue to have utility in unvaccinated individuals or those who respond poorly to vaccination.

We recently described a monoclonal Ab (mAb), isolated from the memory B cells of a SARS survivor obtained 10 years after recovery, that neutralizes SARS-CoV-2 and SARS-CoV through recognition of the S receptor–binding domain (RBD) but without blocking ACE2 attachment (*21*). An optimized version of this mAb (named S309) is currently under evaluation in phase 2/3 clinical trials. The isolation of many other RBD-targeted neutralizing Abs from COVID-19 convalescent patients (*22*–*28*) and the demonstration that they provide in vivo protection against SARS-CoV-2 challenge in small animals and nonhuman primates (*25*, *29*–*31*) showed that the RBD is the major target of neutralizing Abs upon natural CoV infection. Clinical evaluation of therapeutic Abs directly interfering with ACE2 binding is ongoing (*30*–*34*). mAbs with exceptionally high neutralization potency, along with distinct and complementary mechanisms of action compared to existing mAbs, may enable the formulation of mAb cocktails with enhanced efficacy to control the spread of the virus and prevent resistance. Here, we assessed the possibility of combining two ultrapotent neutralizing Abs that we discovered, namely S2E12 and S2M11, which exploit different mechanisms of action.

## Results

### Isolation of ultrapotent SARS-CoV-2 neutralizing Abs

**Fig. 1:**
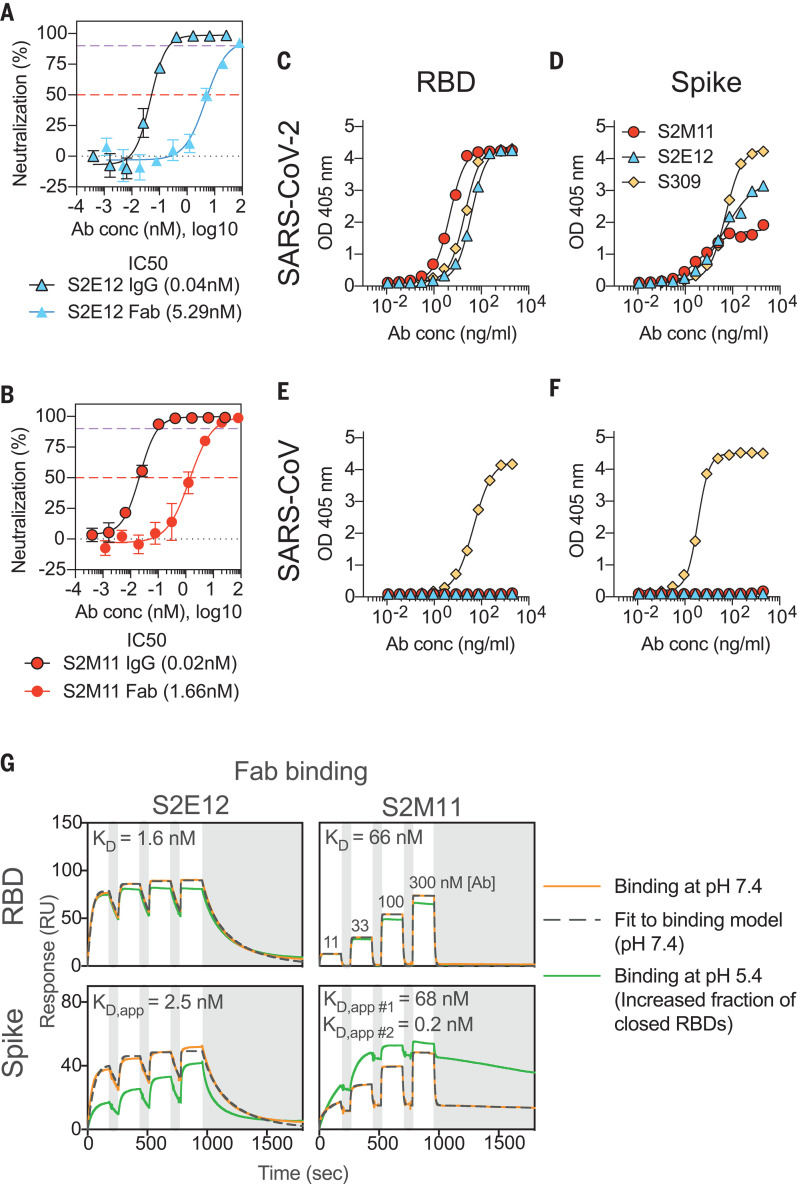
S2E12 and S2M11 neutralize SARS-CoV-2 ultrapotently by targeting the RBD. (**A** and **B**) Neutralization of authentic SARS-CoV-2 (SARS-CoV-2-Nluc) by S2E12 (A) and S2M11 (B) IgG or Fab. Symbols are means ± SD of triplicates. Dashed lines indicate IC_50_ and IC_90_ values. Average IC_50_ values are indicated in parentheses below the graphs (determined from two independent experiments). (**C** to** F**) ELISA binding of S2M11 (red), S2E12 (blue), or S309 (yellow) mAbs to immobilized SARS-CoV-2 RBD (C), SARS-CoV-2 S (D), SARS-CoV RBD (E), or SARS-CoV S (F). Symbols show means of duplicates. (**G**) SPR analysis of S2E12 and S2M11 Fab binding to the SARS-CoV-2 RBD or S ectodomain trimer. Experiments were carried out at pH 7.4 (orange) and pH 5.4 (green) and were repeated twice with similar results (one experiment is shown). The apparent equilibrium dissociation constants (*K*_D, app_) at pH 7.4 are indicated. White and gray stripes indicate association and dissociation phases, respectively. S2M11 binding to S was fit to two parallel kinetic phases and the resulting *K*_D, app #1_ and *K*_D, app #2_ were interpreted as apparent affinities for open RBDs (tertiary epitope) and closed RBDs (quaternary epitope), respectively. This is supported by the similar binding kinetics and affinity of the faster off-rate phase (*K*_D, app #1_) with that observed for S2M11 binding to the isolated RBD (compare with table S1 for full fit results). Ab conc, mAb concentration.

To identify highly potent mAbs elicited upon SARS-CoV-2 infection, we sorted memory B cells from two individuals recovering from severe COVID-19 disease, using biotinylated prefusion SARS-CoV-2 S ectodomain trimer as bait. Two mAbs, S2E12 and S2M11, stood out for their high neutralization activity against authentic SARS-CoV-2 virus and two different SARS-CoV-2 S pseudotyped viruses [using either murine leukemia virus (MLV) or vesicular stomatitis virus (VSV) backbones]. In an assay that measures inhibition of authentic SARS-CoV-2 entry (SARS-CoV-2-Nluc (*35*)), we determined half-maximal inhibitory concentrations (IC_50_) of 3 to 6 ng/ml (20 to 40 pM) (Fig. 1, A and B). We determined IC_50_ values of 1.9 to 2.5 ng/ml for SARS-CoV-2 S-VSV (fig. S1A) and 10.3 to 30.4 ng/ml for SARS-CoV-2 S-MLV (fig. S1B). In an authentic SARS-CoV-2 focus reduction neutralization test that measures inhibition of virus entry and spread (*36*), the IC_50_ values were 1.2 to 6.6 ng/ml (fig. S1C). The exceptional potency of these mAbs was demonstrated further by the concentrations necessary to inhibit 90% of authentic SARS-CoV-2-Nluc viral entry (IC_90_), which we determined as 26.4 ± 7.8 ng/ml and 12.7 ± 3.1 ng/ml for S2E12 and S2M11, respectively (Fig. 1, A and B). The higher neutralization potency of immunoglobulin G (IgG) compared to Fab observed for each mAb suggested that the distinct binding affinities and/or bivalent binding contribute to potency (Fig. 1, A and B). The S2E12 heavy chain uses VH1-58*01, D2-15*01, and JH3*02 genes, whereas S2M11 derives from VH1-2*02, D3-3*01, and JH4*02 genes. The heavy-chain variable gene nucleotide sequence germline identity is 96.53% for S2M11 and 97.6% for S2E12, showing a low level of somatic hypermutation for these two mAbs.

Both S2E12 and S2M11 bound to the SARS-CoV-2 RBD and prefusion-stabilized S ectodomain trimer (*6*) but not to the SARS-CoV RBD or S (*37*) by enzyme-linked immunosorbent assay (ELISA) (Fig. 1, C to F). Using surface plasmon resonance (SPR) and flow cytometry, we further observed that S2E12 and S2M11 compete for binding to the SARS-CoV-2 RBD or to SARS-CoV-2 S, presented either as a recombinantly expressed prefusion-stabilized S ectodomain trimer or as full-length S expressed at the surface of ExpiCHO cells (fig. S2, A and B). When added first, S2M11 competed in a concentration-dependent manner with the sarbecovirus-neutralizing S309 mAb for binding to SARS-CoV-2 S, whereas it could bind with minimal competition when added after S309 (fig. S2B). Whereas the S2E12 Fab (or IgG) bound to SARS-CoV-2 S and RBD similarly, the binding affinity of the S2M11 Fab (or IgG) for the S trimer was enhanced relative to that of the isolated SARS-CoV-2 RBD (Fig. 1G and fig. S2C). Specifically, S2M11 binding kinetics to SARS-CoV-2 S were biphasic, including a first phase with identical binding kinetics and affinity as measured for binding to the isolated RBD, and a second phase with a much slower off-rate and therefore higher affinity. We observed that binding of S2M11 Fab and IgG to S was increased at pH 5.4, a condition that favors the closed trimer conformation, compared to pH 7.4 (*38*) (Fig. 1G, fig. S2C, and table S1). Conversely, binding of the S2E12 Fab to S was diminished at pH 5.4 (and moderately reduced for S2E12 IgG), possibly due to the increased number of S trimers with closed RBDs (Fig. 1G; fig. S2, A and C; and table S1).

Collectively, these findings indicate that S2E12 and S2M11 target overlapping or partially overlapping SARS-CoV-2 RBD epitopes. The finding that S2M11 preferentially interacts with the S trimer relative to the RBD suggests that this mAb might bind to a quaternary epitope only displayed in the context of a native closed prefusion S. Finally, the enhanced binding of S2E12 to SARS-CoV-2 S in conditions favoring RBD opening (pH 7.4) indicates that this mAb might recognize a cryptic epitope not exposed in the closed S trimer.

### S2E12 potently neutralizes SARS-CoV-2 by targeting the RBM

**Fig. 2:**
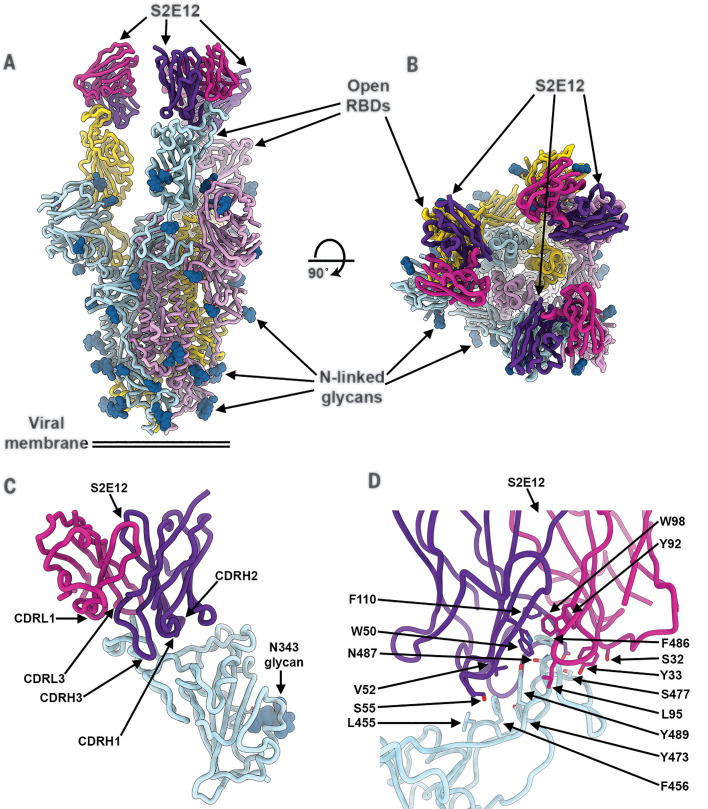
The S2E12 neutralizing mAb recognizes the SARS-CoV-2 RBM. (**A** and **B**) Cryo-EM structure of the prefusion SARS-CoV-2 S ectodomain trimer with three S2E12 Fab fragments bound to three open RBDs viewed along two orthogonal orientations. (**C**) The S2E12 concave paratope recognizes the convex RBM tip. (**D**) Close-up view showing selected interactions formed between S2E12 and the SARS-CoV-2 RBD. In (A) to (D), each SARS-CoV-2 S protomer is colored distinctly (cyan, pink, and gold), whereas the S2E12 light- and heavy-chain variable domains are colored magenta and purple, respectively. N-linked glycans are rendered as blue spheres in (A) to (C). Abbreviations for the amino acid residues are as follows: E, Glu; F, Phe; I, Ile; L, Leu; N, Asn; Q, Gln; S, Ser; T, Thr; V, Val; W, Trp; and Y, Tyr.

To understand the mechanism of S2E12-mediated potent neutralization of SARS-CoV-2, we characterized a complex between the SARS-CoV-2 S ectodomain trimer and the S2E12 Fab fragment using cryo–electron microscopy (cryo-EM). Three-dimensional (3D) classification of the data showed the presence of S trimers with one, two, or three Fabs bound to open RBDs for which we determined structures at 3.5, 3.3, and 3.3-Å resolution, respectively (Fig. 2, A and B; fig. S3, A to G; and table S2). We subsequently used local refinement to obtain a 3.7-Å map of the region corresponding to the S2E12 variable domains and RBD, which markedly improved local resolution due to conformational dynamics relative to the rest of the S trimer, and used it along with a 1.4-Å crystal structure of the S2E12 Fab to build a model (fig. S3, D to G, and tables S2 and S3).

S2E12 recognizes an RBD epitope overlapping with the RBM (i.e., ACE2 receptor-binding site) that is partially buried at the interface between protomers in the closed S trimer (Fig. 2, A to D, and fig. S4, A and B). As a result, S2E12 can only interact with open RBDs, as is the case for ACE2 as well as for several previously described neutralizing mAbs, including S2H14 (*22*, *25*, *28*). The concave S2E12 paratope recognizes the convex RBM tip through electrostatic and van der Waals interactions (Fig. 2, C and D). Specifically, S2E12 utilizes the heavy-chain complementary-determining regions (CDRs) 1 to 3 and the light-chain CDR1 and CDR3, respectively, accounting for two-thirds and one-third of the paratope buried surface area, to recognize residues 455 to 458 and 473 to 493 of the SARS-CoV-2 RBD (Fig. 2, C and D). Nearly all the S2E12 contacts with the RBD are mediated by germline-encoded residues with only one out of five heavy-chain (G109) and one out of four light-chain (G94) mutated residues contributing to the paratope. The structural data explain that S2E12 binds efficiently to both the RBD and the prefusion S trimer (Fig. 1G) and efficiently neutralizes SARS-CoV-2 (Fig. 1, A and B, and fig. S1, A and C): (i) S2E12 recognizes a tertiary 3D epitope, i.e., an epitope that is fully contained within one S protomer; (ii) ~50% of S trimers naturally harbor one open RBD at the viral surface or in recombinantly expressed S ectodomain trimers as observed by cryo–electron tomography and single-particle cryo-EM, respectively (*6*, *39*); and (iii) S2E12 binding shifts the RBD conformational equilibrium toward open S trimers, as previously described for RBM-targeted mAbs (*22*, *28*, *37*).

### S2M11 locks the SARS-CoV-2 S trimer in the closed state through binding to a quaternary epitope

**Fig. 3:**
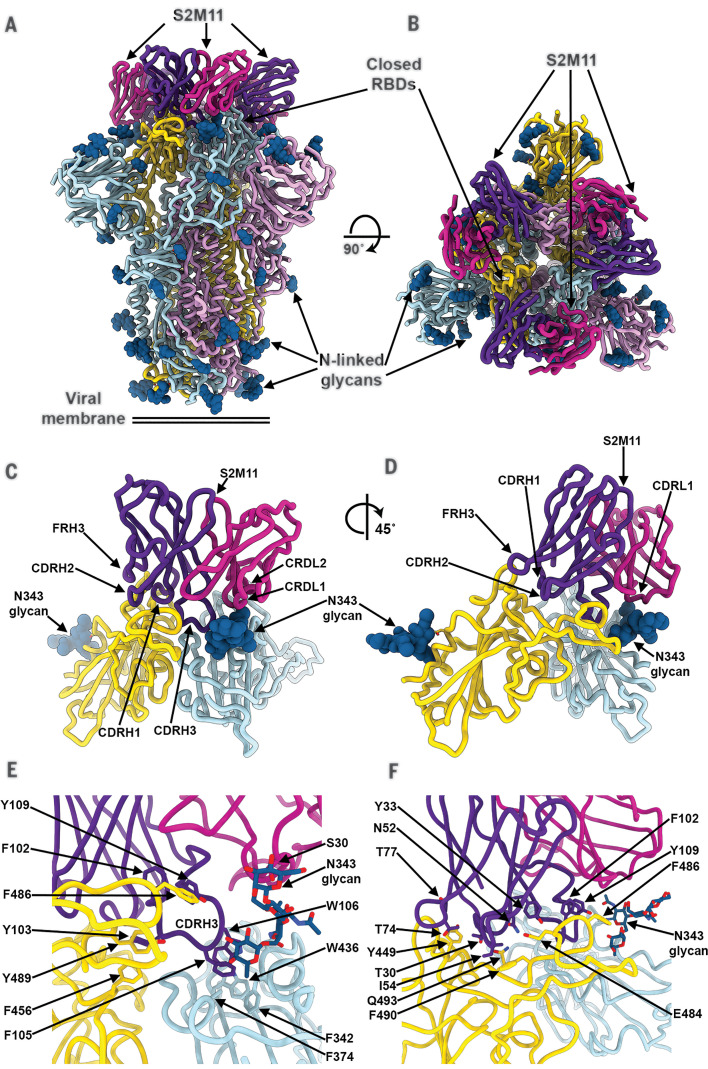
The S2M11 neutralizing mAb recognizes a quaternary epitope spanning two RBDs and stabilizes S in the closed state. (**A** and **B**) Cryo-EM structure of the prefusion SARS-CoV-2 S ectodomain trimer bound to three S2M11 Fab fragments viewed along two orthogonal orientations. (**C** and **D**) The S2M11 binding pose, which involves a quaternary epitope spanning two neighboring RBDs. (**E** and **F**) Close-up views showing selected interactions formed between S2M11 and the SARS-CoV-2 RBDs. In (A) to (F), each SARS-CoV-2 S protomer is colored distinctly (cyan, pink, and gold), whereas the S2M11 light- and heavy-chain variable domains are colored magenta and purple, respectively. N-linked glycans are rendered as blue spheres in (A) to (D) and as sticks in (E) and (F). FR, framework.

We carried out cryo-EM analysis of S2M11 in complex with SARS-CoV-2 S to elucidate the molecular basis of its preferential recognition of the S trimer compared to the RBD and its mechanism of neutralization. Three-dimensional classification of the cryo-EM data revealed the exclusive presence of S trimers adopting a closed conformation, which allowed us to determine a 2.6-Å structure of SARS-CoV-2 S bound to three S2M11 Fab fragments (Fig. 3, A and B; fig. S5, A to F; and table S2). S2M11 recognizes a quaternary epitope through electrostatic interactions and shape complementarity, comprising distinct regions of two neighboring RBDs within an S trimer (Fig. 3, C and D). Specifically, S2M11 CDRH1, CDRH2, and the heavy-chain framework region 3 (FR3) are docked into the RBM crevice (burying a surface of ~400 Å^2^), whereas CDRH3 spans the interface between the RBM and helices 339 to 343 and 367 to 374, as well as residue 436 of an adjacent RBD belonging to the neighboring protomer (i.e., burying a total surface of ~500 Å^2^) (Fig. 3, C and F). Although most interactions are mediated by the S2M11 heavy chain, CDRL2 interacts with residues 440 to 441 and CDRL1 forms key contacts with the glycan at position N343, which is rotated ~45° compared to the orientation that it adopts in the S309-bound S structure (*21*), both sets of interactions occurring with the neighboring RBD (quaternary epitope) (Fig. 3, C and F, and fig. S5G). Three out of eight S2M11 heavy-chain residues that are mutated relative to contribute to epitope recognition (Ile^54^, Thr^77^, and Phe^102^), whereas none of the two light-chain mutated residues participate in RBD binding.

The observation that all particle images correspond to closed S trimers when bound to S2M11 contrasts with our previous finding of ~50%/50% of trimers closed or with one RBD open in the absence of bound mAb (*6*) or in complex with S309 (*21*) or S2H13 (*28*), which do not select for any specific RBD conformation. On the basis of these data, we conclude that S2M11 stabilizes the closed conformation of the S trimer by interacting with a composite epitope including two neighboring RBDs (from two distinct protomers) that are close to each other in the closed state but spread apart upon RBD opening (*6*) (fig. S4, C and D). These results also explain the enhanced S2M11 binding affinity for S compared to the RBD (Fig. 1G), as only the S trimer enables binding to the quaternary epitope, which buries a ~60% greater paratope surface area compared to binding to the isolated RBD (Fig. 3, A to F). We therefore interpret the biphasic binding as S2M11 interacting with a tertiary epitope present in open RBDs (fast off-rate), based on the identical kinetics and affinity measured relative to those of the isolated RBD, and S2M11 recognizing its full quaternary epitope (slow off-rate).

### S2M11 and S2E12 inhibit SARS-CoV-2 attachment to ACE2 and trigger Fc-mediated effector functions

**Fig. 4:**
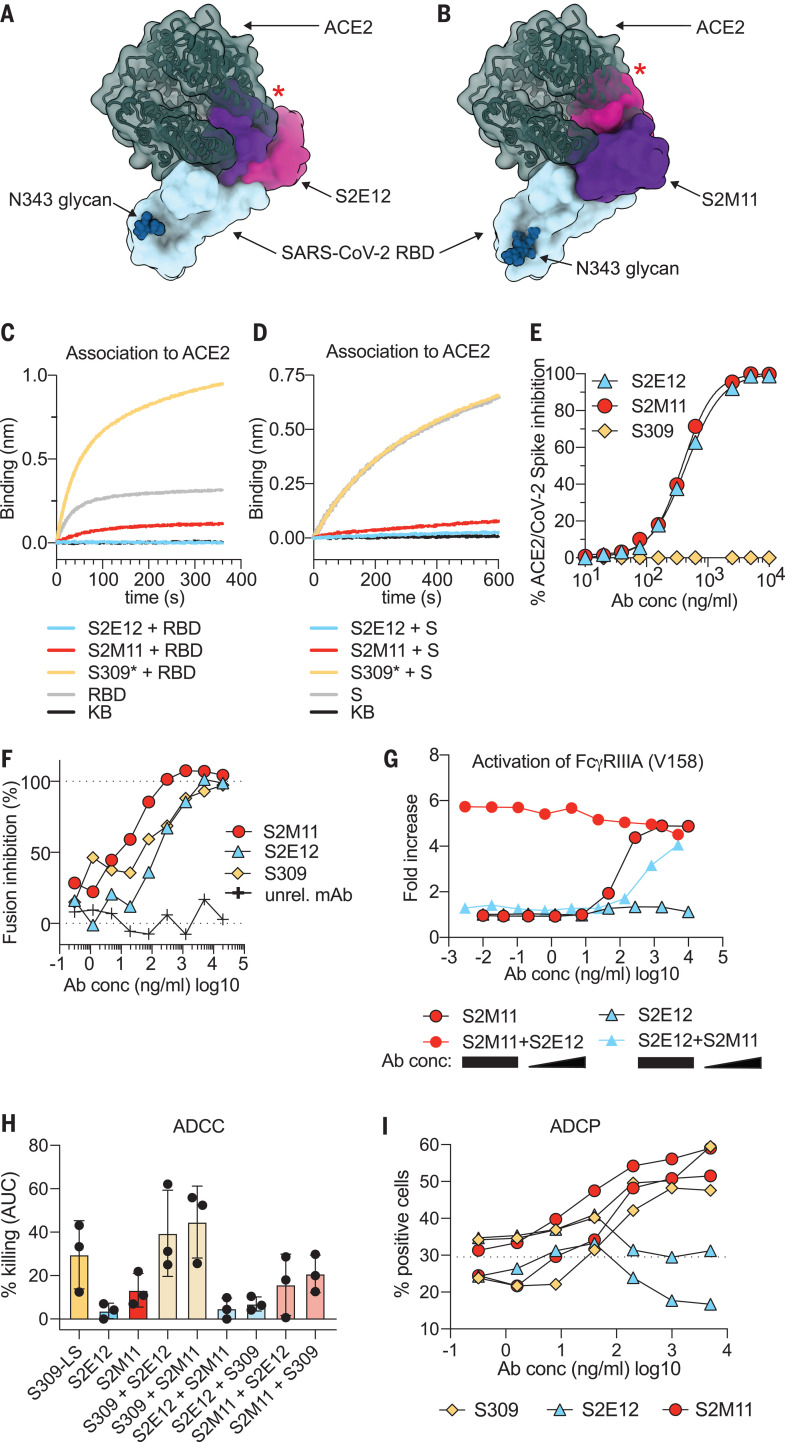
S2E12 and S2M11 prevent SARS-CoV-2 S attachment to ACE2 and inhibit membrane fusion, and S2M11 triggers effector functions. (**A**) S2E12 (magenta/purple) and ACE2 (dark green) bind overlapping binding sites on the SARS-CoV-2 RBD (blue). (**B**) S2M11 (magenta/purple) and ACE2 (dark green) bind overlapping binding sites on the SARS-CoV-2 RBD (blue). The red stars indicate steric clashes. (**C** and **D**) Binding of the SARS-CoV-2 RBD (C) or S ectodomain trimer (D) alone (gray) or precomplexed with the S2M11 (red), S2E12 (blue), or S309* (yellow) mAbs to the ACE2 ectodomain immobilized at the surface of biosensors analyzed by biolayer interferometry. S309* is an optimized version of the parent S309 mAb (*21*). KB, kinetic buffer (negative control). (**E**) Binding of varying concentrations of S2E12 (blue), S2M11 (red), or S309 (yellow) mAbs to full-length S expressed at the surface of CHO cells in the presence of the ACE2 ectodomain (20 μg/ml) analyzed by flow cytometry (one measurement per condition). (**F**) Cell-cell fusion inhibition assay with Vero E6 cells transfected with SARS-CoV-2 S and incubated with varying concentrations of S2E12 (blue), S2M11 (red), S309 (yellow), or a control mAb. The values are normalized to the percentage of fusion without mAb and to the percentage of fusion of nontransfected cells. (**G**) FcγRIIIa (high-affinity variant V158) signaling induced by individual mAbs or mAb cocktails. For mAb cocktails, the concentration of the constant mAb was 5 μg/ml. The concentration of the diluted mAb is indicated on the *x* axis. (**H**) ADCC using primary NK cells as effectors and SARS-CoV-2 S-expressing CHO cells as targets. The magnitude of NK cell–mediated killing is expressed as the area under the curve (AUC) for each mAb used at concentrations ranging between 0.1 ng/ml and 20 μg/ml. For mAb cocktails, the mAb listed first was kept constant at 5 μg/ml. Each symbol represents one donor; data are combined from two individual experiments. See fig. S6E for curves from a representative donor. (**I**) ADCP using peripheral blood mononuclear cells (PBMCs) as a source of phagocytic cells (monocytes) and PKH67–fluorescently labeled S-expressing CHO cells as target cells. The *y* axis indicates percentage of monocytes double-positive for anti-CD14 (monocyte) marker and PKH67. The dashed line indicates the signal detected in the presence of target and effector cells but without mAb (baseline). Each line indicates the data for one PBMC donor. Symbols are means of duplicates. Data are from one experiment. Ab conc, mAb concentration.

The structural data indicate that both S2E12 and S2M11 would compete with ACE2 attachment to the RBD, as they recognize epitopes overlapping with the RBM (Fig. 4, A and B). Moreover, S2M11-induced stabilization of SARS-CoV-2 S in the closed conformational state yields S trimers with masked RBMs that are incompetent for receptor engagement, as previously shown for an engineered S construct covalently stabilized in the closed state (*40*). Hence, both S2E12 and S2M11 blocked binding of SARS-CoV-2 S or RBD to immobilized human recombinant ACE2 measured by biolayer interferometry (Fig. 4, C and D). Additionally, both S2E12 and S2M11 inhibited binding of ACE2 to SARS-CoV-2 S expressed at the surface of Chinese hamster ovary (CHO) cells (Fig. 4E), validating this mechanism of neutralization using full-length native S trimers. The comparable efficiency of S2E12 and S2M11 in blocking S attachment to ACE2 correlates with their similar neutralization potencies.

To further investigate the mechanism of SARS-CoV-2 inhibition by S2E12 and S2M11, we performed a cell-cell fusion assay using VeroE6 cells (which endogenously express ACE2 at their surface) transiently transfected with full-length wild-type SARS-CoV-2 S. Although S2E12 and S2M11 bind and stabilize different conformations of the S protein, both mAbs efficiently blocked syncytia formation (Fig. 4F), which results from S-mediated membrane fusion. The absence of syncytia formation likely is explained by S2E12- or S2M11-mediated disruption of ACE2 binding along with S2M11-induced inhibition of membrane fusion through conformational trapping of SARS-CoV-2 S in the closed state.

Ab-dependent cell cytotoxicity (ADCC) mediated by natural killer cells or Ab-dependent cell phagocytosis (ADCP) mediated by macrophages or monocytes are Fc-mediated effector functions that can contribute to protection by facilitating virus clearance and by supporting immune responses in vivo*,* independently of direct neutralization (*41*). As a prerequisite for ADCC to occur, we first demonstrated that infected cells express SARS-CoV-2 S on their surface (fig. S6, A and B). Then, to evaluate the ability of S2M11 and S2E12 to leverage ADCC and ADCP, we tested if these mAbs (IgG1 backbone) could induce FcγRIIa and FcγRIIIa-mediated signaling using a luciferase reporter assay. S2M11 promoted efficient, dose-dependent FcγRIIIa-mediated (but not FcγRIIa-mediated) signaling, in particular for the high-affinity (V158) variant of the Fc receptor, to levels comparable to that of the cross-reactive mAb S309 (Fig. 4G and fig. S6, C and D) (*21*). By contrast, S2E12 triggered FcγRIIa-mediated (but not FcγRIIIa-mediated) signaling, possibly as a result of the distinct orientation of the mAb relative to the membrane of the effector cells in comparison to S2M11 and S309 (Fig. 4G and fig. S6C). Accordingly, S2M11 but not S2E12 showed FcγRIIIa-dependent ADCC activity (Fig. 4H and fig. S6E) and ADCP activity (Fig. 4I). As we observed efficient activation of effector functions when mixing S2M11 with S2E12 or S309 (Fig. 4, G and H, and fig. S6E), we propose that cocktails of these mAbs can leverage additional protective mechanisms in vivo besides inhibition of viral entry.

### Formulation of ultrapotent neutralizing Ab cocktails against SARS-CoV-2

Surveillance efforts have led to the identification of a number of S mutants among circulating SARS-CoV-2 isolates. Several naturally occurring RBD mutations were shown to abrogate interactions with known mAbs and to reduce immune sera binding, raising concerns that viral neutralization escape mutants could emerge or be selected under pressure from mAb-based antiviral treatments (*42*). To investigate if S2E12- and S2M11-mediated neutralization might be affected by SARS-CoV-2 polymorphism, we tested binding of either mAb to 29 S protein variants (corresponding to mutations detected in circulating SARS-CoV-2 isolates) expressed at the surface of Expi CHO cells. The Y449N, E484K/Q, F490L, and S494P RBD variants led to decreased S2M11 binding to S, whereas none of the mutants tested affected interactions with S2E12, although several of them are found in the epitope of this latter mAb (table S4). The impact of these substitutions on S2M11 binding is explained by the structural data showing that the SARS-CoV-2 S Y449 and E484 side chains are hydrogen-bonded to the S2M11 heavy-chain F29 backbone amide and the N52/S55 side chains, respectively, and the F490 and S494 residues are buried at the interface with S2M11. SARS-CoV-2 S-VSV pseudotyped virus entry assays with selected S variants confirmed these results and showed that the Y449N, E484K/Q, F490L/S, and S494P individual substitutions abrogated S2M11-mediated neutralization, whereas the L455F variant reduced neutralization potency by an order of magnitude (fig. S7, A, C, and E). S2E12 neutralized efficiently all variants tested except G476S that showed an order-of-magnitude decreased activity (fig. S7, B, D, and F). In agreement with deep mutational scanning data (*43*), we found that the Y449N variant was impaired in its ability to bind ACE2 (fig. S8), which is expected to reduce viral fitness, likely explaining that this mutation has been reported to date in only one out of 90,287 complete SARS-CoV-2 genome sequences. Although rare, the G476S, E484K/Q, S494P, and F490L/S mutations have been detected in 20, 10 (E to K) or 17 (E to Q), 15, and 5 (F to L) or 8 (F to S) viral isolates, respectively, and in theory could be selected under the selective pressure of S2E12 or S2M11. Overall, 15 SARS-CoV-2 S variants with a single amino acid substitution within the S2M11 epitope were reported, with a prevalence of less than 0.1% as of September 2020 (fig. S7G).

To circumvent the risk of emergence or selection of neutralization escape mutants, we assessed whether S2M11, S2E12, and S309 could be combined in two-component mAb cocktails on the basis of their complementary mechanisms of action. SARS-CoV-2 S-VSV pseudotyped virus entry assays showed that mAb cocktails potently neutralized the Y449N, S494P, and G476S variants and overcame the neutralization escape phenotype observed with single mAbs (fig. S7, H to J). A concentration matrix of S2E12 and S2M11 revealed their additive neutralization effects without antagonism, even though both Abs compete for binding to the RBM (fig. S9, A to C). Moreover, the combination of S309 with S2E12, which do not compete for binding to S, and S309 and S2M11, which partially compete (i.e., for attachment to the closed S trimer), also yielded additive neutralization effects (fig. S9, D to F), suggesting that two- (or three-) component mAb cocktails are a promising therapeutic strategy to prevent the emergence or the selection of viral mutants escaping mAb therapy.

### S2M11 and S2E12 protect hamsters against SARS-CoV-2 challenge

**Fig. 5:**
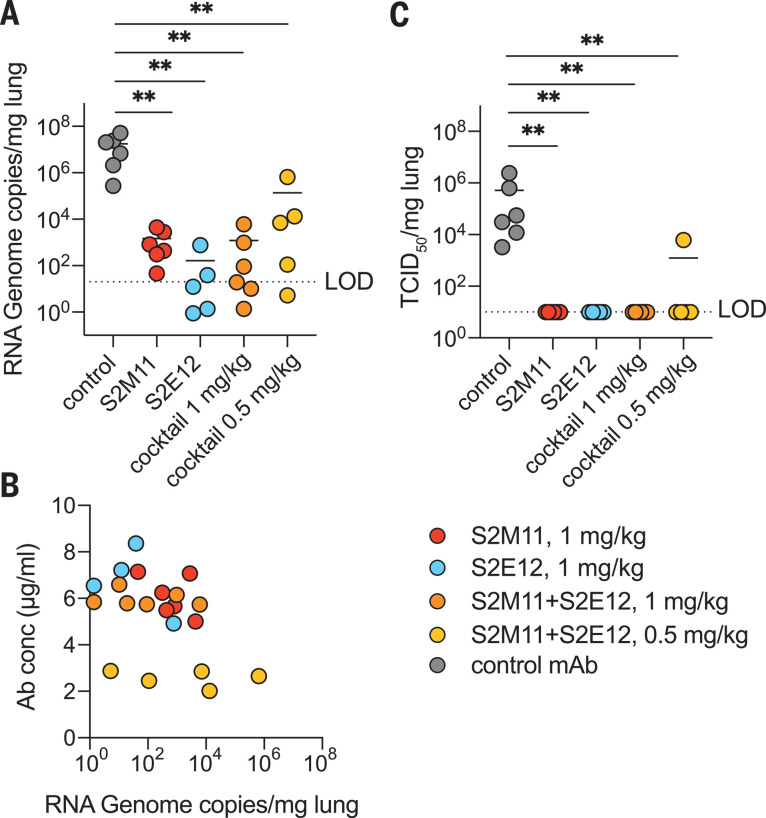
S2E12, S2M11, or cocktails of the two mAbs provide robust in vivo protection against SARS-CoV-2 challenge. Syrian hamsters were injected with the indicated amount of mAbs 48 hours before intranasal challenge with SARS-CoV-2. (**A**) Quantification of viral RNA in the lungs 4 days after infection. (**B**) The concentration of mAbs measured in the serum before infection (day 0) inversely correlates with the viral RNA load in the lung 4 days after infection. (**C**) Quantification of replicating virus in lung homogenates harvested 4 days after infection using a TCID_50_ assay. For mAb cocktails, the total dose of an equimolar mixture of both mAbs is indicated.

To evaluate the protective efficacy of S2E12 and S2M11 against SARS-CoV-2 challenge in vivo, we tested either mAb or a cocktail of both mAbs in a Syrian hamster model (*44*). The mAbs were engineered with heavy- and light-chain constant regions from Syrian hamster IgG2 to allow optimal triggering of Fc-dependent effector functions. mAbs were administered by intraperitoneal injection 48 hours before intranasal challenge with 2 × 10^6^ median tissue culture infectious dose (TCID_50_) of SARS-CoV-2. Four days later, lungs were collected for the quantification of viral RNA and infectious virus. Either mAb alone or cocktails with 0.5 mg/kg or 1 mg/kg total mAb decreased the amount of viral RNA detected in the lungs by two to five orders of magnitude compared to hamsters receiving a control mAb (Fig. 5A). The amounts of viral RNA detected at day 4 inversely correlated with serum mAb concentration measured at the time of infection (Spearman’s R −0.574, *p* = 0.0052) (Fig. 5B). Prophylactic administration of these mAbs at all doses tested completely abrogated viral replication in the lungs, with the exception of a single animal that received the low-dose cocktail and was partially protected (Fig. 5C). These data show a notable protective efficacy of both mAbs at low doses, individually or as cocktails, in line with their ultrapotent in vitro neutralization.

## Discussion

S2M11 and S2E12 were identified among almost 800 screened Abs isolated from 12 individuals who recovered from COVID-19. The ultrapotency and quaternary epitope of S2M11 appear to be rare compared to more canonical RBM-specific neutralizing Abs, as the latter type of mAbs were present in every donor we analyzed. A mAb recognizing the closed S conformation (mAb 2-43) was previously identified, and low-resolution mapping of its binding site suggested that it might interact with a quaternary epitope that appears distinct from that of S2M11 (*45*). Two recent reports describe the identification of a mAb and of a nanobody targeting quaternary epitopes, spanning two neighboring RBDs, which are present in the closed S trimer. Nb6 was identified from a naïve nanobody library, affinity matured and trimerized to achieve an IC_50_ of 160 pM, however, without the ability to exert effector functions (*46*). C144 was isolated from a COVID-19 convalescent serum sample, uses *VH3-53* and *VL2-14* genes, harbors a 25-residue long CDRH3, and efficiently neutralizes SARS-CoV-2 (*47*). Similar to S2M11, Nb6 (along with its engineered derivatives) and C144 use CDR(H)3 to bridge two neighboring RBDs and stabilize SARS-CoV-2 S in the closed state. A long CDRH3 of 15 or more amino acid residues was a common feature of C144-type mAbs (*47*). Contrary to the C144 25-residue-long CDRH3, S2M11 achieves this bridging with a relatively short CDRH3 of 18 amino acids [IMGT definition (*48*)]. As a result, all three binders inhibit SARS-CoV-2 through interfering with ACE2 attachment to S through direct competition and locking of the S trimer in the closed state. mAbs recognizing viral surface glycoproteins by binding to quaternary epitopes have been identified against Epstein-Bar virus (*49*), dengue virus (*50*–*53*), Zika virus (*54*), Ebola virus (*55*), West Nile virus (*56*), and HIV (*57*) and proved to be exceptionally potent or broad. S2M11, along with Nb6 and C144, therefore defines a distinct class of potent neutralizers of SARS-CoV-2 relative to previously isolated mAbs.

We recently described that the magnitude of Ab responses to SARS-CoV-2 S and nucleoprotein and neutralizing Ab titers correlate with clinical scores (*28*). The SARS-CoV-2 RBD is the main target of potent neutralizing S-specific Abs in COVID-19 patient sera or plasma samples, thereby focusing most of the selective pressure imposed by the humoral immune response on this domain (*23*, *28*). Given that several RBD variants have been found among circulating SARS-CoV-2 isolates, combining RBD-specific mAbs with different binding modes and distinct mechanisms of neutralization could prove essential for successful clinical application. A combination of S2M11 and S2E12 or cocktails of either of these mAbs with S309 yielded additive effects on neutralization potency. Moreover, Ab cocktails comprising S309 and/or S2M11 demonstrated robust activation of ADCC and ADCP, suggesting that combining these mAbs using distinct neutralization mechanisms would trigger these protective mechanisms in vivo. S2E12 and S2M11 (harboring a hamster Fc), individually or formulated as cocktails, conferred significant protection using mAb doses that are, to our knowledge, the lowest reported for human mAbs tested in hamster models. As a result, the mAb cocktails characterized here are expected to take advantage of both ultrapotent neutralization, different mechanisms of action, and Fc-mediated effector functions to protect from a broad spectrum of circulating SARS-CoV-2 isolates and limit the emergence of neutralization escape mutants. We propose that combinations of mAbs leveraging multiple distinct mechanisms of action with additive or synergistic effects could provide additional benefits for clinical application.

## Supplementary Materials

science.sciencemag.org/content/370/6519/950/suppl/DC1 Materials and Methods Figs. S1 to S9 Tables S1 to S4 References (*58*–*83*) MDAR Reproducibility Checklist

## Appendix

View/request a protocol for this paper from *Bio-protocol*.
